# Type B Aortic Dissection Masquerading As Acute Pyelonephritis: Think Beyond Measures

**DOI:** 10.7759/cureus.54343

**Published:** 2024-02-17

**Authors:** Anas Ibraheem, Abdullah Abdullah, Kumari Priyam, Rebin Fakhruddin

**Affiliations:** 1 Internal Medicine, Imamein Kadhimein Medical City, Baghdad, IRQ; 2 Internal Medicine/Clinical Hematology, Al Karama Teaching Hospital, Baghdad, IRQ; 3 General Medicine, Frimley Health NHS Foundation Trust, Wexham Park Hospital, Slough, GBR; 4 Acute Medicine, Pilgrim Hospital, United Lincolnshire Hospitals Trust, Boston, GBR

**Keywords:** case report, awareness, hypertension, vascular emergencies, atypical presentation, diagnostic challenge, kidney infarction, acute pyelonephritis, type b aortic dissection, aortic dissection

## Abstract

Aortic dissection (AD) is a life-threatening medical emergency with a high mortality rate if misdiagnosed; therefore, an urgent and precise diagnosis is crucial for prompt treatment. This article presents a rare case report of AD with an atypical clinical presentation that led to delayed diagnosis and a complicated clinical course. Herein, we aim to contribute to the existing literature by providing insights into the varied presentations of AD and offering valuable lessons for clinicians faced with similar diagnostic scenarios. A 64-year-old female with an extended history of hypertension and other comorbidities presented to the emergency department with a one-day duration of right-sided loin pain and fever. Her blood investigations demonstrated evidence of leukocytosis and high c-reactive protein (CRP) levels. She was preliminarily treated as a case of acute pyelonephritis since, initially, clinical and radiographic evidence did not yield an alternative diagnosis. Despite antibiotics, her condition deteriorated, and her urine output became less than 0.5 mL/kg/hour for six consecutive hours. Additionally, the obtained urine culture was negative on the third day of admission, which made the medical team repeat her history taking and clinical examination, revealing a previously overlooked weight loss. This red flag prompted the medical team to conduct thorough chest and abdominal imaging studies in search of any hidden malignancy, especially when her thyroid function test returned normal. Surprisingly, a contrast-enhanced abdominal CT scan demonstrated an infarcted right kidney by thromboembolism that originated from the partially obstructive thrombus in the proximal abdominal aorta, which was later confirmed to be a type B AD by a CT angiogram. A multidisciplinary team guided her treatment, which included carefully controlling her blood pressure, using anticoagulants, and closely monitoring the patient. The take-home messages of this case report underscore the critical importance of recognizing atypical clinical presentations of AD, overcoming diagnostic challenges through comprehensive approaches, tailoring treatments to individual patient needs, and advocating for a multidisciplinary and patient-centered approach to enhance overall clinical outcomes.

## Introduction

Have you ever thought about the possibility of aortic dissection (AD) when creating your list of differential diagnoses? This condition is often overlooked due to its broad and unpredictable presentation [1]. It occurs when a tear develops in the inner lining of the aorta. The tear allows blood to enter and separate the space between the aortic intima and media, creating a false lumen by increasing intraluminal pressure [1,2].

AD is the most common aortic emergency that typically affects older men with a long history of hypertension. This makes the combination of mechanical factors and degenerative changes a reasonable pathogenesis behind AD, as discussed by Sayed A et al. [3]. Although it can occur in individuals without known risk factors, hypertension is the most common threat. Smoking, high cholesterol, pre-existing or familial aortic diseases, aortic valve disease, history of cardiac surgery, direct and blunt chest trauma, and intravenous drug use are also associated with this condition. Additionally, inherited diseases like Marfan's syndrome and Ehlers-Danlos syndrome can increase the risk of developing dissections [1,2].

Two conventional systems classify AD based on the disease's anatomical extent. The Stanford system categorizes dissections into two types. Type A affects any part of the ascending aorta proximal to the brachiocephalic artery. In contrast, type B arises distal to the left subclavian artery origin and only affects the descending aorta. The DeBakey system classifies dissections based on the location of the intimal tear. Type I involves a tear in the ascending aorta that extends to the aortic arch, type II has a tear only in the ascending aorta, and type III has a tear in the descending aorta [1,2]. The proposed type-entry-malperfusion (TEM) classification by Sievers HH et al. offers improved outcome prediction and clarity of the disease's anatomical and clinical extent; however, further studies are needed for validation [4].

In this study, we highlight a 64-year-old female with unusual clinical manifestations of AD that led to a delayed diagnosis and a complicated clinical course. Join us as we explore the importance of recognizing such a case scenario with all the difficulties in achieving a prompt diagnosis. This case aims to contribute to the existing literature by providing insights into the varied presentations of AD and offering valuable lessons for clinicians in similar diagnostic scenarios to think beyond measures for enhanced patient care.

## Case presentation

A 64-year-old female with a past medical history of hypertension, chronic obstructive pulmonary disease (COPD), fibromyalgia, anxiety disorder, and cephalosporin allergy arrived at the emergency department complaining of sudden, sharp, and continuous right flank pain with no radiation, which was present for the last 24 hours. It was associated with chills, fever, nausea, non-bloody vomiting, and one episode of loose bowel movement on the day of admission. Additionally, she stated that her pain was a nine on a scale of 0-10, where eight and above indicated severe pain. Her systematic review was unremarkable: no cough, shortness of breath, chest pain, or lower urinary tract symptoms of dysuria, hematuria, frequency, or nocturia. Regarding her social history, she was a one-pack-year smoker and a heavy alcohol drinker. She lived with her son and was usually independent.

Upon examination, she was altered and oriented to time, place, and person, with a noticeable fine hand tremor, suggesting alcohol withdrawal symptoms. Her vital signs were as follows: blood pressure of 160/109 mmHg of both arms, pulse rate of 112 beats per minute without a deficit, respiratory rate of 20 cycles per minute, temperature of 37.8°C, and oxygen saturation of 92% on ambient air. Her abdominal examination was unremarkable except for right-sided lower quadrant tenderness with absent rebound tenderness, psoas, Rovsing’s, obturator, and McBurney’s signs. However, a right-sided costophrenic angle tenderness was elicited. It is worth noting that she had no signs of lymphadenopathy. Her initial blood investigations revealed leukocytosis (18.4 × 10^9^/L), a high c-reactive protein (CRP) level (77 mg/L), and a slightly elevated serum lactate level (1.2 mmol/L). However, other measured parameters were normal, including renal function test and venous blood gas analysis (Table 1). Her general urine test showed hematuria of eight RBC/high power field (HPF) and pyuria of 10 WBC/HPF. A CT scan of the kidneys, ureters, and bladder (CT KUB) without contrast was sufficient to exclude acute appendicitis, pancreatitis, and renal stones (Video 1).

**Table 1:** Summary of the patient’s laboratory data on admission ALP, alkaline phosphatase; ALT, alanine aminotransferase; AST, aspartate aminotransferase; BUN, blood urea nitrogen; Cl, chloride; CRP, C-reactive protein; K, potassium; MCH, mean corpuscular hemoglobin; MCHC, mean corpuscular hemoglobin concentration; MCV, mean corpuscular volume; Na, sodium; RDW, red cell distribution width

Test	Result	Reference range	Test	Result	Reference range
White blood count	18.4 × 10^9^/L	4.5-11× 10^9^/L	Na	139 mmol/L	135-145 mmol/L
Neutrophils	16.3 K/µL	2-7 K/µL	K	4.0 mmol/L	3.5-5 mmol/L
Lymphocytes	0.6 K/µL	1.5-4 K/µL	Cl	97 mmol/L	96-106 mmol/L
Monocytes	1.4 K/µL	0.2-0.8 K/µL	Albumin	41 g/dL	34-54 g/L
Eosinophils	0.1 K/µL	0.01-0.04 K/µL	ALP	116 U/L	35-104 U/L
Basophils	0.0 K/µL	<0.01 K/µL	ALT	11 U/L	10-35 U/L
Platelet count	237 × 10^9^/L	145-400 × 10^9^/L	AST	85 U/L	10-35 U/L
Red blood cell count	4.76 × 10^12^/L	3.8-5.8 × 10^12^/L	Total bilirubin	19 µmol/L	1.71-20.5 µmol/L
Hemoglobin level	14.5 g/dL	11.5-16 g/dL	BUN	4.2 mg/dL	5-20 mg/dL
MCV	95.2 fL	80-100 fL	Creatinine	66 µmol/L	53-97.2 µmol/L
MCH	30.5 pg	27-32 pg	Amylase	29 U/L	30-110 U/L
MCHC	32 g/dL	32-36 g/dL	CRP	77 mg/dL	0.3-1.0 mg/dL
RDW	15.9%	12-15%	Serum lactate	1.2 mmol/L	<1.0 mmol/L

**Video 1:** A CT KUB shows a sizeable uncomplicated gallstone (yellow arrow) and a calcified abdominal aorta (red arrow), indicating subclinical atherosclerotic disease. CT KUB, CT scan of the kidneys, ureters, and bladder

**Figure 1:**
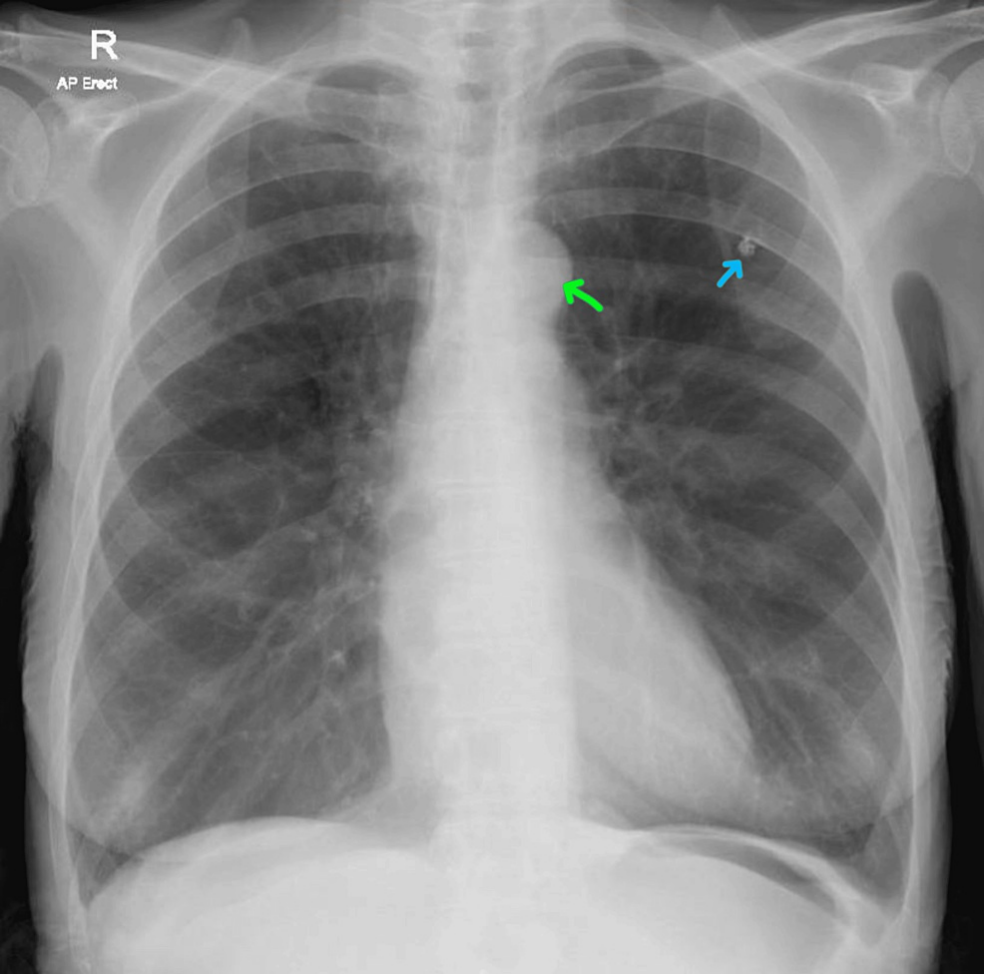
An erect chest X-ray shows lung hyperinflation with a flattened diaphragm consistent with chronic obstructive pulmonary disease, a calcified granuloma at the left upper zone (blue arrow), and aortic knob enlargement (green arrow) consistent with her long history of hypertension. AP, anteroposterior

On the other hand, her chest X-ray revealed features consistent with COPD and a calcified granuloma in the left upper lung field, which was under a three-month serial surveillance (Figure 1). Her electrocardiogram was insignificant. In addition, the real-time reverse-transcriptase polymerase-chain-reaction (rRT-PCR) test was negative for COVID-19. Therefore, she was preliminarily treated as a case of acute pyelonephritis with empirical intravenous antibiotics (gentamicin at first, then changed to ciprofloxacin) and supportive measures, including morphine for pain relief. The treatment was given while awaiting the results of her urine culture and sensitivity test for potentially further guidance and treatment.

Surprisingly, the urine culture returned negative three days later, as well as a step-ladder rise in her white blood count (28.7 × 10^9^/L) and CRP (295 mg/dL and 368 mg/dL on days two and three, respectively). Her monitoring charts showed a decreasing trend in urine output (<0.5 mL/kg/hour for six consecutive hours) with an elevation of serum creatinine up to 78 µmol/L on the third day of admission. Upon repeating the history and clinical examination, it was discovered that she had unintentionally lost 7.4 kg of body weight since her last dietetic review six months ago, which was not previously noted. The patient's unexplained weight loss prompted the medical team to conduct thorough chest and abdominal imaging studies in search of any hidden malignancy, especially when her thyroid function test returned normal. A chest CT scan revealed nothing significant except for the calcified granuloma, which exhibited no malignant features. On the other hand, the contrast-enhanced abdominal CT scan demonstrated a complete blockage of the right renal artery by thromboembolism that originated from a partially obstructive thrombus in the proximal abdominal aorta (Video 2). Unfortunately, the right kidney was entirely infarcted and beyond repair, and the urology team decided against surgical intervention.

**Video 2:** A contrast-enhanced abdominal CT scan demonstrates a complete blockage of the right renal artery by thromboembolism, resulting in right kidney infarction (blue arrow), originating from the partially obstructive thrombus in the proximal abdominal aorta (yellow arrow). The left kidney is normally enhanced (green arrow).

**Figure 2:**
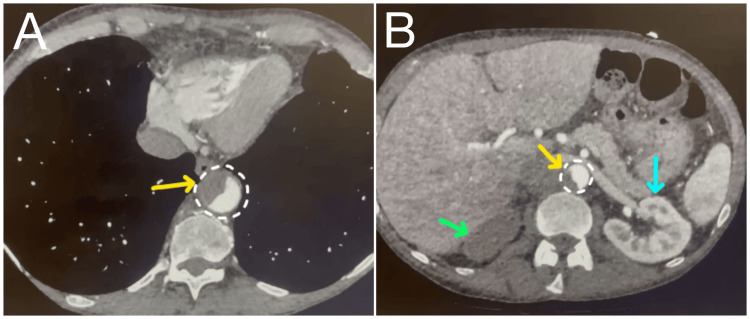
A CT angiogram of the chest (A) and abdomen (B) sections shows a partially occluded and dissected aorta (demarcated by a circle and pinpointed with yellow arrows) from T10-L3 level, confirming type B aortic dissection. The right kidney is infarcted (green arrow), while the left one is normally enhanced (blue arrow).

A CT angiogram (CTA) confirmed the presence of a type B aortic dissection (TBAD) with a thrombosed false lumen (Figure 2). A multidisciplinary team comprising a urologist, a cardiovascular surgeon, and an internist decided on a treatment plan that involved antibiotic escalation, administering intravenous labetalol with a systolic target of less than 120 mmHg, initiating anticoagulants to counteract thrombosis, and performing serial imaging. Additionally, they emphasized the need for continuous monitoring of her vital signs, urine output, signs of abdominal pain, the lower limbs’ neurovascular function, and arterial blood gas analysis every four hours. Despite the final diagnosis being made, the weight loss remained a mystery. The dietician attributed that to the patient's past medical history, which included early stages of alcohol-related liver disease, anxiety disorder, and fibromyalgia, which could have been the reason for poor dietary consumption and increased metabolic rate. Therefore, she was offered a high-protein and high-calorie diet.

The antihypertensive regimen comprised an initial dose of 20 mg administered over two minutes, followed by additional increments of 20-40 mg at 10-minute intervals until the desired blood pressure was achieved. Maintenance infusion at a rate of 2 mg/minute was subsequently employed to sustain the target blood pressure. Her condition remained stable after 72 hours, except for a new-onset diarrhea, which was later confirmed positive for clostridium difficile. As a result, her antibiotic was changed from co-amoxiclav (1.2 g) to fidaxomicin (200 mg) and gentamicin (100 mg). The anticoagulation team advised continuing on subcutaneous dalteparin (200 IU/kg/day) until the international normalized ratio (INR) reaches two. After almost a month, the patient's condition improved as her pain and fever gradually subsided. She also regained her usual urine output and bowel habits. The white blood count and CRP levels returned to normal, while the serum creatinine level stabilized at around 65 µmol/L. The serial images demonstrated a reduced thrombus size. Consequently, she was discharged on warfarin (10 mg/day) with an initial follow-up visit at the hospital clinic two days later and later on by her general practitioner for long-term surveillance. In terms of her daily activities, she was advised to avoid heavy lifting to prevent strain or discomfort and to refrain from overexertion during aerobic activities, aiming for 30 minutes of mild to moderate aerobic exercise on most days of the week.

## Discussion

AD presenting symptoms lack consensus, mainly due to the various organs that the aorta supplies along its path. These organs can be affected eventually by either the aortic tear per se or the subsequent complications of the occlusive thromboembolism, leading to features that overlap or mimic those of other diseases [1,2,5]. For instance, paraplegia may occur due to the involvement of spinal arteries, abdominal pain and change in bowel motion may result from mesenteric artery involvement, limb ischemia due to distal aortic involvement, stroke due to carotid artery involvement, and acute kidney injury due to renal artery involvement [2,6,7]. It is worth mentioning that the cardiovascular system is not an exception; either myocardial ischemia causes chest pain, arrhythmia, and other symptoms due to coronary artery involvement, or aortic root involvement with aortic regurgitation, resulting in features of congestive heart failure and pleural effusions. Upon examination, such patients typically have a pulse deficit and a difference in blood pressure in the limbs, comparing both the right and left sides of the body [2,5,8].

Regardless, pain is the most reliable historical symptom of AD, based on its prevalence and reflective nature [6]. Firstly, it correlates to the location of the underlying pathology. Ascending AD commonly presents as anterior chest or neck and jaw pain. In contrast, pain in the back and abdomen suggests descending aortic pathology. Secondly, it suddenly reaches its maximum intensity compared to other differentials, like acute myocardial infarction, which starts slowly and gradually increases in intensity over time. Thirdly, the pain caused by AD can be migratory in nature as the condition progresses. Thus, the patient may experience upper chest pain that eventually proceeds down to the lower chest or upper abdomen. Although AD is typically associated with tearing pain, it is more commonly described as sharp rather than tearing, ripping, or stabbing [2,9].

Numerous studies have pointed out the challenges associated with accurate and timely diagnosis of AD [10-12]. The rate of misdiagnosis during the first evaluation in the emergency department can be as high as 78%, especially for patients who present as 'walk-in' patients upon hospital admission [5]. In our patient, right-sided loin pain with fever and evidence of leukocytosis and high CRP levels can be easily misdiagnosed as acute pyelonephritis, especially when initial clinical and radiographic evidence failed to provide an alternative diagnosis. In such case scenarios, pyrexia can be misleading because pyrogens can be released from the injured aortic wall, leading to a false indicator of underlying infection [13].

The diagnosis of AD is often unrecognized and probably missed because of the highly variable clinical presentation, making it a challenging clinical problem [10,11]. According to Lovatt S et al., around one in three patients with AD are misdiagnosed due to the presence of symptoms that are associated with other diseases, as well as the absence of typical features like a pulse deficit and widened mediastinum on chest X-ray [5]. This diagnostic dilemma was seen in our patient. In 2010, the American Heart Association and the American College of Cardiology collaborated to create the Aortic Dissection Detection Risk Score (ADD-RS) to reduce misdiagnosis of AD syndrome and minimize over-testing. The score consists of three categories: predisposing conditions, pain features, and physical findings. If the overall score is >1, it is advisable to consider a CTA. If it is ≤1, consider a D-dimer test. If the D-dimer is <500 ng/mL, consider a chest X-ray to search for a widened mediastinum, or if the D-dimer is ≥500 ng/mL, consider CTA [12].

Repeating history taking and clinical examination was the best next step for our patient. This revealed that she had experienced weight loss, which was missed during the initial clerking. Although a contrast-enhanced abdominal CT scan was performed to check for possible malignancies, it actually revealed something unexpected. The patient was found to have a complicated thrombus in the right renal artery, which had infarcted her right kidney. The irony is that if intravenous contrast had been used in the initial CT KUB, the diagnosis of AD could have been made on the first day of admission. Lovatt S et al. suggested that a more comprehensive history taking and increased imaging use can lead to a more accurate diagnosis of AD [5]. Therefore, a CTA was carried out, which confirmed the presence of TBAD. This test is considered the gold standard for diagnosing AD, being 97% sensitive and 100% specific [11,14].

In another reality, unlike the one she was approached as acute pyelonephritis, she might have initially been assessed against the ADD-RS at the emergency department. It would have been found that she had a score of ≤1 related to her severe abrupt loin pain, indicating the need for a D-dimer assessment. That said, in this reality, if the ADD-RS had been assessed initially and the D-dimer was ≥500 ng/mL, unnecessary over-testing could have been avoided, and much time and effort could have been saved. Unfortunately, the patient was not assessed using this approach, and an initial D-dimer was not evaluated; therefore, routine use of ADD-RS should be encouraged among medical professionals, even in a pre-clinical setting.

The therapeutic approach for TBAD can be summarized as follows: closed patient monitoring for possible complications alongside aggressive control of blood pressure with a systolic target between 100 and 120 mmHg [11,15]. Although recent studies have shown better outcomes with lower morbidity and mortality rates after thoracic endovascular aortic repair (TEVAR) or open repair (OR), these surgical interventions are still recommended for only severely complicated TBAD [2,11,16]. Regarding anticoagulant use, different studies in the literature demonstrate the suitability and apparent safety of anticoagulants in the setting of acute aortic syndrome [17,18]. However, current guidelines lack recommendations for anticoagulant use, particularly in type B AD, and advise for patient-tailored therapy until findings from larger studies are available [19,20]. As part of our decision-making process for the patient's treatment, we prioritized moral values like autonomy, beneficence, non-maleficence, and justice alongside current guidelines to weigh the pros and cons of anticoagulant use and avoidance of surgical intervention. Despite the medical advancements, AD has a high mortality rate of approximately 1% per hour after initial diagnosis, and it can reach up to 50% by the third day for untreated cases; therefore, a high index of suspicion is crucial [2].

## Conclusions

This case emphasizes the significance of recognizing an unusual clinical presentation of AD and the challenges it may pose for prompt diagnosis and management. Proper history-taking, meticulous physical examination, and increased use of imaging techniques can improve the accuracy and speed of detection. Routine use of ADD-RS in pre-clinical settings should be encouraged as it increases the likelihood of obtaining a correct diagnosis. Controlling blood pressure and tailoring treatment to the patient's needs can significantly improve the overall outcome.
